# Native, Memory-Bounded Conversion of Mass Spectrometry Imaging Data into the SpatialData Ecosystem

**DOI:** 10.1021/jasms.6c00169

**Published:** 2026-09-22

**Authors:** Theodoros Visvikis, Wouter-Michiel Vierdag, Luca Marconato, Angeliki Birmpili, Ron M.A. Heeren, Eva Cuypers

**Affiliations:** † The Maastricht MultiModal Molecular Imaging (M4I) Institute, Division of Imaging Mass Spectrometry (IMS), 5211 Maastricht University, Maastricht 6229 ER, The Netherlands; ‡ European Molecular Biology Laboratory, Genome Biology Unit, Heidelberg 69117, Germany

## Abstract

Mass spectrometry imaging (MSI) is now a routine modality for spatial molecular profiling and is increasingly combined with histology, multiplexed imaging, and spatial transcriptomics to build richer descriptions of tissue than any single technique can provide. The conceptual case for this multimodal paradigm was settled a decade ago, but the data infrastructure needed to operationalize it at scale has not kept up. Vendor binaries and the imzML exchange format keep MSI outside the OME-NGFF, Zarr, and SpatialData stacks on which the rest of spatial omics has converged, leaving each new experiment to rebuild ad hoc converters from scratch. Here, we present Thyra, an open-source Python library that converts raw vendor and imzML MSI data directly into the SpatialData format. A two-pass streaming sparse matrix engine writes the spectral matrix straight into the output Zarr store, keeping peak memory bounded near 200 MB regardless of data set size and supporting 100+ GB acquisitions. On a representative mouse brain MALDI data set, this yields three- to 4-fold storage reductions and an order-of-magnitude improvement in *m*/*z* range query latency, the bottleneck for ion image generation. Once converted, MSI data are directly composable with the scverse analysis ecosystem; we quantify this with an scverse readiness benchmark in which spatial autocorrelation, pixel clustering, and neighborhood enrichment, all standard operations in spatial transcriptomics, run as a one-liner on the converted object. A brief exploratory analysis of a skin biopsy from a psoriasis patient illustrates the analytical surface that the unified container exposes.

## Graphical abstract

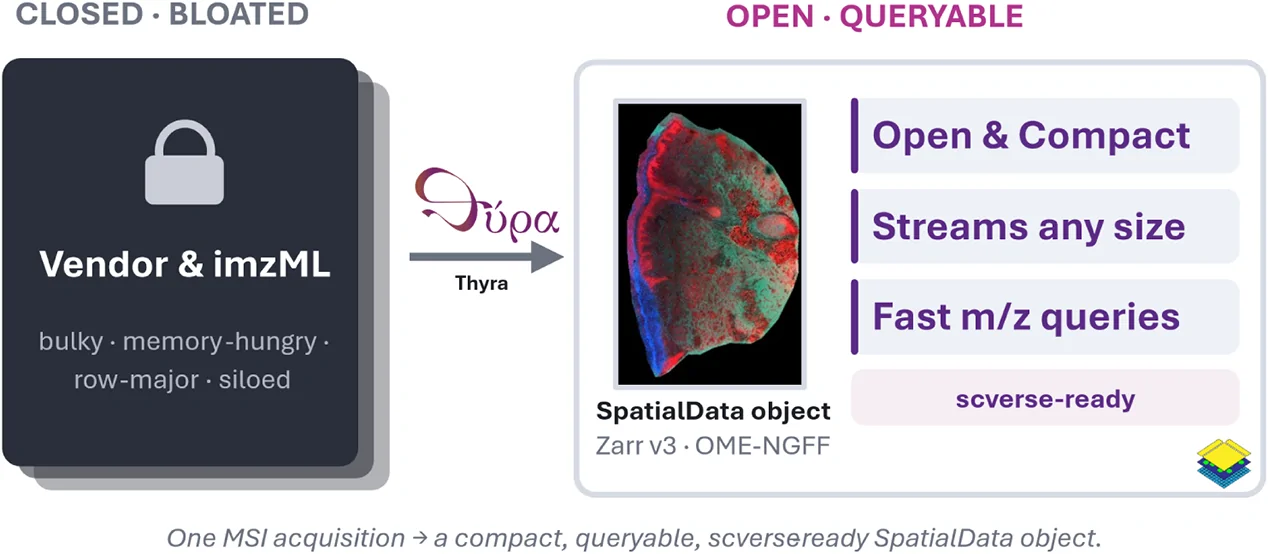

## 1 Introduction

Mass spectrometry imaging (MSI) maps the spatial distributions of molecules across tissues, materials, cultural heritage artifacts, and other surfaces, providing untargeted information across thousands of molecular channels in a single acquisition.^1,2^ Over the past two decades, the technique has matured from a niche capability into a routine modality across lipidomics, metabolomics, drug-distribution studies, and clinical diagnostics.^3^

A turning point in how the community thought about MSI came when image fusion of MSI with high-resolution microscopy was shown to yield molecular maps with spatial resolution and chemical contrast that neither modality could provide alone.^4^ Subsequent work has extended the multimodal paradigm to immunofluorescence, multiplexed protein imaging, and, increasingly, to spatial transcriptomics, establishing the case that the most informative molecular description of a tissue is rarely the one any single instrument produces.^5^

In parallel, the broader spatial biology community has converged on a shared computational infrastructure for multimodal data. Spatial transcriptomics platforms have moved from proof of concept to commercial systems imaging hundreds of genes at subcellular resolution, and highly multiplexed protein imaging has matured in parallel.^6^ The computational community that grew up around these technologies has standardized on the OME-NGFF specification^7^ for cloud-native bioimaging, the Zarr v3 array format for chunked storage, AnnData^8^ for annotated tabular data, and the SpatialData framework,^9^ part of the scverse ecosystem,^10^ as a unifying container for multimodal spatial data. The result is a substrate in which a histology image, a transcriptomics data set, and a derived feature table can be loaded into a single object, registered to a common coordinate system, and queried analytically without bespoke parsers.

MSI has remained largely outside of this substrate. Vendor data sit in proprietary binary formats whose specifications are partly closed, and the open exchange standard, imzML,^11^ was a major step forward when introduced but predates the demands of modern spatial biology. imzML parses its XML metadata in full at load time, scales poorly with data set size, and lacks lazy loading, columnar slicing, and cloud-native access. The pragmatic consequence is that, even though the conceptual case for multimodal MSI is settled, the workflows needed to realize it remain unique per laboratory and per data set. Each new fusion experiment is rebuilt from scratch: custom converters, manual coordinate handling, ad hoc registration scripts, and the resulting analyses are difficult to reproduce, share, or scale. Existing converters that target the SpatialData ecosystem (e.g., metaspace-converter^12^) primarily retrieve cloud-processed annotations rather than full, continuous raw data, leaving a gap for direct, local conversion of complete vendor imzML data sets.

Here we introduce Thyra (from Greek θúρα, meaning “door”), an open-source Python library that converts raw vendor and imzML MSI data directly into the SpatialData format. By writing into the same multimodal container used by spatial transcriptomics, multiplexed imaging, and bioimaging tools, Thyra removes the format barrier between MSI and the rest of spatial omics. Two design choices set it apart from prior conversion tools: it converts raw, continuous spectra rather than centroided or annotated outputs, enabling de novo signal processing on the converted object; and a two-pass streaming sparse matrix engine writes the spectral matrix directly into the output Zarr store, keeping peak memory bounded regardless of data set size. We report Thyra’s architecture, characterize storage and analytical query performance against vendor raw and processed imzML formats, introduce a scverse-readiness benchmark that quantifies how the choice of substrate affects the analytical operations downstream MSI work most often relies on, and present a brief exploratory analysis on a skin biopsy from a psoriasis patient to illustrate the analytical surface that the unified container exposes. Multimodal extensions, in which MSI is jointly registered with paired histology or transcriptomics, follow naturally once registration tooling is in place; the present work establishes the foundation on which those extensions will compose. A companion manuscript (under review) introduces Thyra to the spatial-omics community and sets out this multimodal vision; here we focus on the conversion engine, its performance, and its analytical composability for mass spectrometry imaging. The complete end-to-end conversion and analysis workflow is summarized in Figure 1.

**Figure 1:**
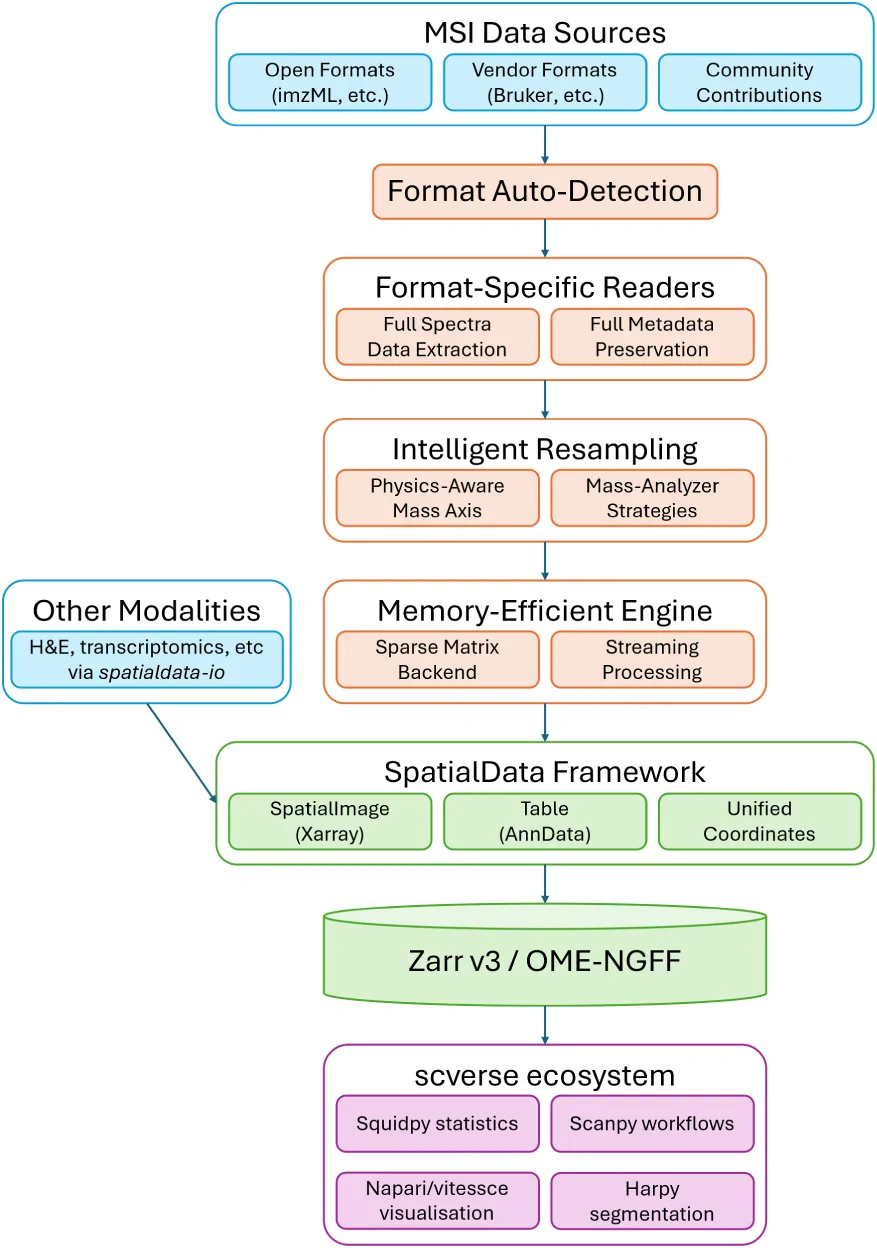
Overview of the end-to-end MSI data integration workflow. Diverse MSI inputs, including open (e.g., imzML) and proprietary vendor formats, are ingested via automated format detection to extract full spectra and metadata. The data undergo intelligent resampling using physics-aware strategies tailored to the mass analyzer type, handled by a memory-efficient engine that utilizes sparse matrix backends and streaming processing. Additional spatial modalities (e.g., H&E, transcriptomics) can be integrated via spatialdata-io. The processed data are structured into the SpatialData model—incorporating SpatialImage (Zarr array), Table (AnnData), and unified coordinates—and stored using the cloud-optimized Zarr v3/OME-NGFF standard. This standardization enables downstream analysis and visualization within the scverse ecosystem using tools such as Squidpy,^13^ Scanpy,^14^ Napari,^15^ Vitessce,^16^ and Harpy.^17^.

## 2 Materials and Methods

### 2.1 Software Architecture

Thyra is implemented as a modular, plugin-based Python library. A thread-safe registry performs automatic format detection and dispatches input files to the appropriate reader plugin (imzML, Bruker.d, Waters.raw, via the vendors’ respective SDKs, with additional vendor formats supported through community-contributed plugins inheriting from a shared base class). All plugins inherit from standardized abstract base classes (BaseMSIReader and BaseMSIConverter), allowing new formats to be added without modifying the core code base. The package is distributed under the MIT license and is managed by using Poetry for reproducible dependency resolution. The conversion core has a deliberately minimal dependency footprint: SpatialData is the only spatial-omics package required, and no downstream analysis libraries (for example, Squidpy or Scanpy) are needed to convert data, so users who want only the Zarr/SpatialData output can install and run Thyra without the full analysis stack. Dependency version bounds are pinned to avoid transitive resolution conflicts that can otherwise arise in fresh environments.

### 2.2 Spectral Harmonization

Spectral harmonization is governed by a resampling decision tree that parses instrument metadata to choose between nearest-neighbor resampling for centroided data and total-ion-current-preserving interpolation for profile data. The system supports physics-aware nonlinear axes for the relevant analyzer geometries, preserving the native sampling characteristics of the instrument.

Resampling projects every spectrum onto a single common *m*/*z* axis, whose bin spacing is derived from the instrument’s mass-resolving power rather than from a fixed bin width (Figure 2). For analyzers whose peak width scales with *m*/*z*, such as TOF, FT-ICR, and Orbitrap, the axis is nonlinear: the local bin spacing at each *m*/*z* is set to a fixed fraction of the expected peak full width at half-maximum (fwhm) at that *m*/*z*, computed from the resolving power recorded in the acquisition metadata. This keeps the number of samples across a peak constant over the whole range, so mass accuracy is preserved uniformly, rather than oversampling low *m*/*z* and under-sampling high *m*/*z*, as a fixed-width grid would. The common mass axis is generated with instrument-appropriate bin spacing, set by a target bin width specified at a reference *m*/*z* and propagated across the mass range according to the analyzer’s resolution physics (for time-of-flight data, for example, the bin width scales as √(*m*/*z*)). By default, this reference width is 0.017 Da at *m*/*z* 300 for linear-TOF data and 0.005 Da at *m*/*z* 1000 for reflector-TOF, Orbitrap, and FT-ICR data; it can be decreased to retain finer detail in very high-resolution spectra or increased to reduce output size.

**Figure 2:**
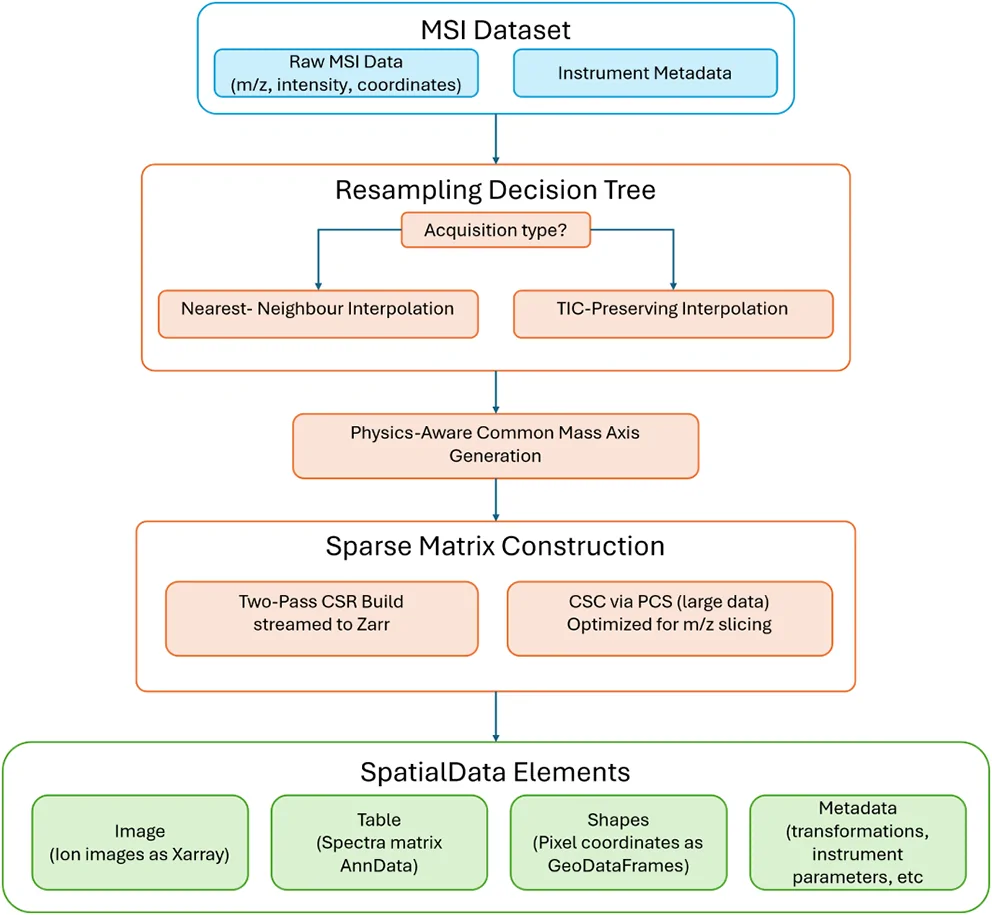
Detailed data processing and resampling workflow.

Intensities are mapped onto this axis by one of two routes selected from the metadata. For profile data, TIC-preserving interpolation assigns each output bin the integral of the source spectrum over the bin interval, so that the total ion current of every pixel is conserved exactly rather than being altered by point resampling. For already centroided data, each peak is assigned to its nearest axis bin, preserving both position and intensity. Because the axis oversamples the native peak width, profile peak shapes are retained, and peak detection performed on the converted object recovers centroids consistent with vendor software peak picking on the same spectra.

### 2.3 Two-Pass Streaming Sparse Engine

To handle data sets larger than RAM, the conversion engine writes the spectral matrix directly into the output Zarr store rather than building an intermediate SciPy matrix in memory. For data sets up to roughly 30 GB, a two-pass streaming pipeline is used. A first pass over the input spectra counts nonzeros per row to lay out the row-oriented sparse index structure (indptr); a second pass streams resampled intensities and their column indices straight to disk in compressed sparse row (CSR) format. The output is then optionally transposed in place to compressed sparse column (CSC) format, the orientation that minimizes latency for the dominant downstream query of retrieving a single *m*/*z* range across every pixel for ion image generation; CSC is the user-facing default, and the format can be selected via the – sparse-format flag. For data sets above the configurable 30 GB threshold, the engine automatically switches to a Precalculated Scatter (PCS) path that uses memory-mapped temporary files to construct the column layout in place, avoiding the two-to-three-fold peak memory spike that a naive coordinate list (COO) build followed by COO-to-CSC conversion would incur. Across both paths, peak-resident memory stays bounded at roughly 200 MB regardless of data set size, so the same conversion command runs unmodified from gigabyte-scale benchmark slides to the 100-GB-class acquisitions typical of modern multimodal experiments.

In the resulting matrix, rows are pixels (observations) and columns are *m*/*z* channels (features). This follows the AnnData and scverse convention, in which observations are rows and variables are columns. Readers more familiar with the Seurat or Bioconductor convention, where the matrix is transposed so that features are rows and cells are columns, should note the difference, because the orientation determines which axis is stored contiguously and therefore which queries are fast. The two compressed layouts make opposite trade-offs on this matrix. Compressed sparse row (CSR) stores each pixel’s full spectrum contiguously and is fastest for retrieving all features of a single pixel or region (per-pixel access). Compressed sparse column (CSC) stores each *m*/*z* channel contiguously and is fastest for retrieving one *m*/*z* range across all pixels, which is the dominant operation in ion image generation. Thyra writes CSC by default for this reason; the orientation can be switched with the *–sparse-format* flag, and downstream tools that iterate pixel-by-pixel may prefer CSR.

### 2.4 SpatialData Mapping and Storage

Harmonized data are written into the SpatialData model: ion images as Xarray-backed SpatialImage elements, the spectral matrix as an AnnData table, pixel coordinates as Shapes mapped to a unified physical coordinate system, and instrument metadata are preserved alongside. Storage uses Zarr v3, which supports sharding and asynchronous I/O for cloud-native access.

Raw MSI data (*m*/*z*, intensity, coordinates) and instrument metadata are routed through a resampling decision tree that selects either nearest-neighbor or total ion count (TIC)-preserving interpolation based on the acquisition type. This is followed by the generation of a physics-aware common mass axis to preserve spectral accuracy. A memory-efficient engine writes the spectral matrix directly into the output Zarr store: a first pass counts the nonzeros per row to lay out a compressed sparse row (CSR) index, a second pass streams intensities and column indices to disk, and the result is optionally transposed to Compressed Sparse Column (CSC) to optimize downstream *m*/*z* slicing, with a precalculated scatter (PCS) path that builds the CSC layout in place for very large data sets. This streaming design avoids an intermediate coordinate list (COO) build. The final output is structured into distinct spatial data elements: image (ion images via Xarray), table (spectral matrix via AnnData), shapes (pixel coordinates via GeoDataFrames), and comprehensive metadata.

### 2.5 Validation

Conversion fidelity is validated through three quantitative metrics: metadata preservation (comparison of instrument parameters between the source and the converted object), data integrity (quantitative comparison of total ion current images), and spectral fidelity (direct comparison of individual mass spectra). Continuous integration comprises unit tests and performance benchmarks that are executed on each commit.

To establish that conversion is correct across instrument platforms and not specific to a single geometry, the three fidelity checks (metadata preservation, total-ion-current image integrity, and per-pixel spectral fidelity) are applied to data from all supported input families: Bruker (.d/.tdf/.tsf, baf), Waters (.raw), and imzML, in both continuous and processed profile and centroid forms. In every case, the converted object reproduces the source metadata, total-ion-current image, and individual spectra. Conversion correctness is the vendor-specific step of the pipeline because each format is read by a dedicated plugin; once data are mapped onto the common-axis SpatialData representation, all inputs share an identical structure, so the downstream storage and query performance reported in Section 3 is a property of that shared representation rather than of any source format. Conversion correctness is additionally enforced continuously by the test suite, which converts a reference file for each supported vendor on every commit.

### 2.6 Benchmark Data Set and Hardware

The storage and analytical query benchmarks reported in Sections 3.1 and 3.2 were performed on a single representative data set, a coronal mouse brain MALDI MSI acquisition (Bruker timsTOF, 18 GB raw, 918,855 pixels), in three formats: the original Bruker.d (vendor raw), a continuous-axis-processed imzML exported from SCiLS, and the Thyra-generated SpatialData/Zarr object. The two analysis-ready formats represent fundamentally different storage strategies: the processed imzML stores data in a ragged-array mode with distinct *m*/*z* axes per pixel, while the SpatialData object stores all spectra against a single common mass axis. All benchmarks ran sequentially on a single thread on an Intel Core i9–14900K (3.20 GHz), 128 GB RAM, with local SSD storage, so the timings reflect format and storage layout rather than parallelism. The exploratory analysis of Section 3.4 is a separate psoriasis skin biopsy described in Section 2.9.

### 2.7 Storage and Analytical Query Benchmark Protocols

Three access patterns were implemented to stress-test data organization. Random pixel access generated 100 random pixel indices: spectra were retrieved via pyimzML for imzML, via SQLite coordinate mapping for Bruker.d (eliminating sequential iteration overhead), and via direct row slicing on the sparse CSC matrix for SpatialData. Random *m*/*z* range queries (100 ranges, widths 100–1,000 bins) simulated ion-image extraction; for imzML and Bruker formats this required iterating through all pixels to retrieve complete spectra, while for SpatialData, columnar slicing (matrix­[:, start:end]) was used. Random ROI extraction generated 100 random rectangular ROIs (10–50-pixel side lengths); spectra within each ROI were retrieved and a mean spectrum computed. All measurements used Python’s time.perf_counter; for lazy-loading formats, computations was forced to produce dense arrays to prevent measurement of only query construction time. Results were aggregated from 100 iterations per pattern, reporting median latencies. Initialization time (file parsing and metadata extraction) was measured separately and excluded from query latencies.

### 2.8 scverse Readiness Benchmark Protocols

Three single-modality analytical operations were performed on the converted SpatialData object using the scverse stack, each chosen to mirror an operation that downstream MSI work routinely requires and that is classically rebuilt from scratch on bespoke data structures. Operation A computed Moran’s I for a panel of candidate ions to identify those with significant spatial autocorrelation across the tissue, using Squidpy’s sq.gr.spatial_autocorr13. Operation B clustered pixels in spectral space, performing PCA followed by Leiden clustering and UMAP embedding via standard Scanpy calls (sc.pp.pca, sc.pp.neighbors, sc.tl.leiden, sc.tl.umap)­14, treating the spectral matrix as if pixels were single cells. Operation C characterized the spatial co-occurrence of pixel clusters using Squidpy’s sq.gr.nhood_enrichment, identifying which spectral populations tend to lie next to each other in tissue space.

The MSI feature matrix used as input to the three operations was prepared at load time, not inside the analytical core. Pixels with zero total ion intensity (empty rectangle from a partial-region export) were dropped, intensities were summarized onto a peak list of 267 peaks via fwhm-sum integration (the sum of all *m*/*z* bins within ± fwhm/2 of each peak *m*/*z*), and the resulting matrix was Pareto-normalized (per-pixel TIC followed by mean-centering and division by the square root of the per-feature standard deviation). Three normalization schemes (TIC, Median, Pareto) were compared on the same peak panel using two spatial-coherence metrics, per-pixel neighbor purity and spatial-graph modularity; Pareto produced the highest scores on both and was used throughout (Supporting Information Figure S2 and Table S1).

To quantify the analytical API overhead reduction enabled by composing on the SpatialData substrate, we counted lines of code (executable Python lines, excluding imports and comments) in the analytical core of each operation. The scverse implementation is compared against a hand-coded scientific Python reference using identical algorithms (Leiden community detection on a Euclidean k-nearest-neighbor graph in PCA space via igraph and leidenalg, the same libraries Scanpy uses internally; Moran’s I and neighborhood enrichment implemented from NumPy and SciPy primitives). We do not report wall-clock timings: the two implementations run the same algorithms on the same preprocessed matrix, so they differ only marginally in runtime. The meaningful difference is code complexity.

### 2.9 Exploratory Analysis Data Set

The exploratory analysis reported in Section 3.4 was performed on a skin biopsy from a psoriasis patient (Bruker timsTOF MALDI in lipid mode, 5-μm pixel size), part of an ongoing study of lipid distributions in psoriatic skin. Of the 424,800 grid pixels in the partial-region export, 291,015 contained tissue, and the remainder were dropped by an empty-pixel filter (TIC = 0) at load time. Spectra were summarized onto a panel of 267 manually curated peaks via fwhm-sum integration, and the resulting matrix was Pareto normalized. The same tissue section was also imaged by Xenium spatial transcriptomics and stained with H&E, with histopathological annotations (epidermis, dermis, sweat gland, hair follicle, stratum corneum, dermal inflammatory infiltrate, orthokeratosis, restored granular layer, mild acanthosis) provided by an expert dermatopathologist. Conversion to SpatialData was performed with the default Thyra settings. All downstream analyses used Squidpy 1.x and Scanpy 1.10.x in a single Jupyter notebook.

## 3 Results

### 3.1 Storage Efficiency

A naive harmonization of the brain test data set to a continuous-axis imzML produced a 455 GB file, prohibitive for routine use. The processed (centroided) imzML file exported from SCiLS was 15 GB. The Thyra-generated SpatialData object was 4.83 GB, smaller than both the original 18 GB Bruker.d file and the 15 GB processed imzML, with no detectable loss in spectral fidelity (verified by direct total ion current and per-pixel spectrum comparison). The reduction is driven by chunked Zarr compression and a sparse columnar layout. Storage results are summarized in Figure 3a.

**Figure 3:**
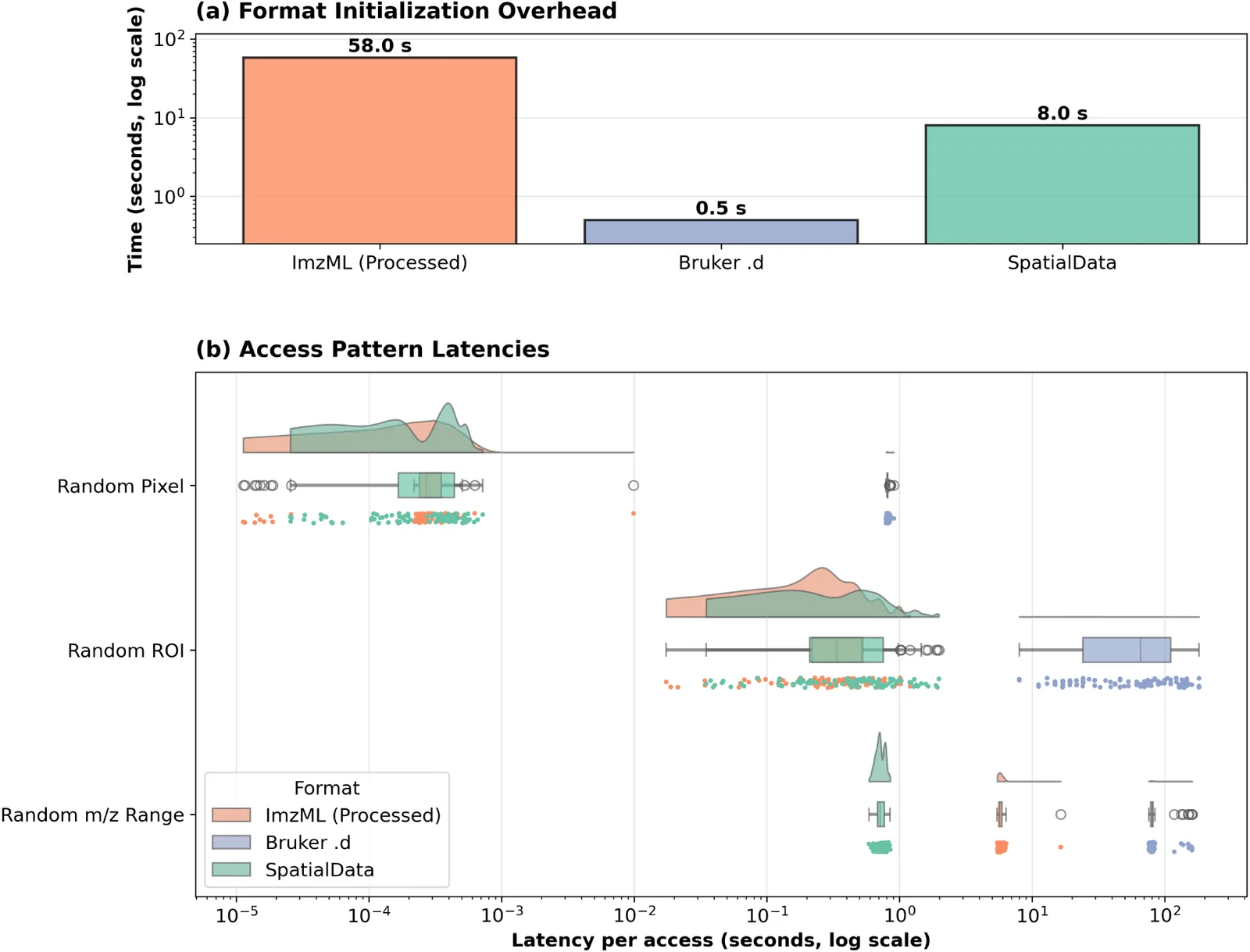
Performance benchmark results for initialization time and query latency. The benchmark compares three MSI data formats: the raw Bruker.d (blue), a processed imzML (orange), and the converted SpatialData object (green). (a) One-time initialization cost (“time to first query”). SpatialData (8.0 s) significantly outperforms imzML (58.0 s) in startup latency, while the raw Bruker format is fastest (0.5 s). (b) Raincloud plots displaying log-scaled query latency for 100 random queries across three distinct access patterns. For random pixel access, imzML retains a slight latency advantage, whereas for random ROI extraction, the performance between imzML and SpatialData is comparable. Crucially, SpatialData achieves an order-of-magnitude speedup for random *m*/*z* range queries, the bottleneck operation for generating ion images. The raw Bruker format shows consistently higher latency across all analytical patterns, confirming its unsuitability for direct downstream analysis.

### 3.2 Initialization and Analytical Query Performance

imzML required 58 s to parse its XML metadata at every session, a recurring cost that compounds across exploratory analysis cycles. The raw Bruker format was the fastest to open (0.5 s) because it does no harmonization. SpatialData incurred a one-time 8 s cost to load the library ecosystem; subsequent sessions on the same data set were effectively free (Figure 3b).

Across 100 random queries per access pattern, the comparison was nuanced (Figure 3c). For random pixel access, imzML retained a small latency advantage for local storage. For ROI extraction, imzML and spatial data were comparable. For the bottleneck operation—random *m*/*z* range queries that drive ion image generation—SpatialData was nearly an order of magnitude faster than processed imzML because columnar Zarr slicing avoids the noncontiguous reads that a row-major imzML layout requires. The raw Bruker format was orders of magnitude slower across all analytical patterns and is not appropriate as a downstream analysis substrate.

### 3.3 scverse Readiness Benchmark

Storage and query latencies describe the substrate but do not measure how the choice of substrate affects the analytical operations that MSI users actually run. To address this, we report a scverse-readiness benchmark consisting of three operations on the converted SpatialData object, each chosen to mirror an analysis classically rebuilt from scratch on bespoke MSI data structures.

Operation A computed Moran’s I across a panel of candidate ions to identify those with significant spatial autocorrelation across the tissue, using Squidpy’s sq.gr.spatial_autocorr. Operation B clustered pixels in spectral space, performing PCA followed by Leiden clustering and UMAP embedding via standard Scanpy calls (sc.pp.pca, sc.pp.neighbors, sc.tl.leiden, sc.tl.umap), treating the spectral matrix as if pixels were single cells. Operation C characterized the spatial co-occurrence of the resulting pixel clusters using Squidpy’s sq.gr.nhood_enrichment, identifying which spectral populations tend to lie next to each other in tissue space.

All three operations expressed the analytical core in substantially fewer lines of code than the hand-coded scientific Python equivalent: 8, 5, and 7 executable lines on the SpatialData object, against 26, 16, and 21 lines for Operations A, B, and C, respectively, a 3.1-fold reduction overall. The savings come from two sources. The substrate carries pixel coordinates and AnnData semantics directly, so there is no glue code to assemble a feature matrix from raw arrays; the scverse stack already implements the spatial statistics and clustering primitives the operations need, so the call sites collapse to a few named functions. The full side-by-side listings are provided in the Code Repository.

### 3.4 Exploratory Analysis on a Skin Psoriasis Data Set

To illustrate the analytical surface that the unified container exposes, we performed a brief exploratory analysis on a skin biopsy from a psoriasis patient (the acquisition described in Section 2.9), using only the operations introduced in Section 3.3. The same tissue section was also imaged by Xenium spatial transcriptomics and stained with H&E for paired histology, with the H&E annotated by an expert dermatopathologist (Figure 4, top row). These paired modalities are shown alongside the MSI as the multimodal acquisition; their joint registration to a common coordinate system is the natural follow-on once registration tooling is restored, at which point the same scverse readiness operations will work across all three modalities without reengineering. The full notebook ran in a few minutes on commodity hardware with no MSI-specific glue beyond the initial Thyra conversion call.

**Figure 4:**
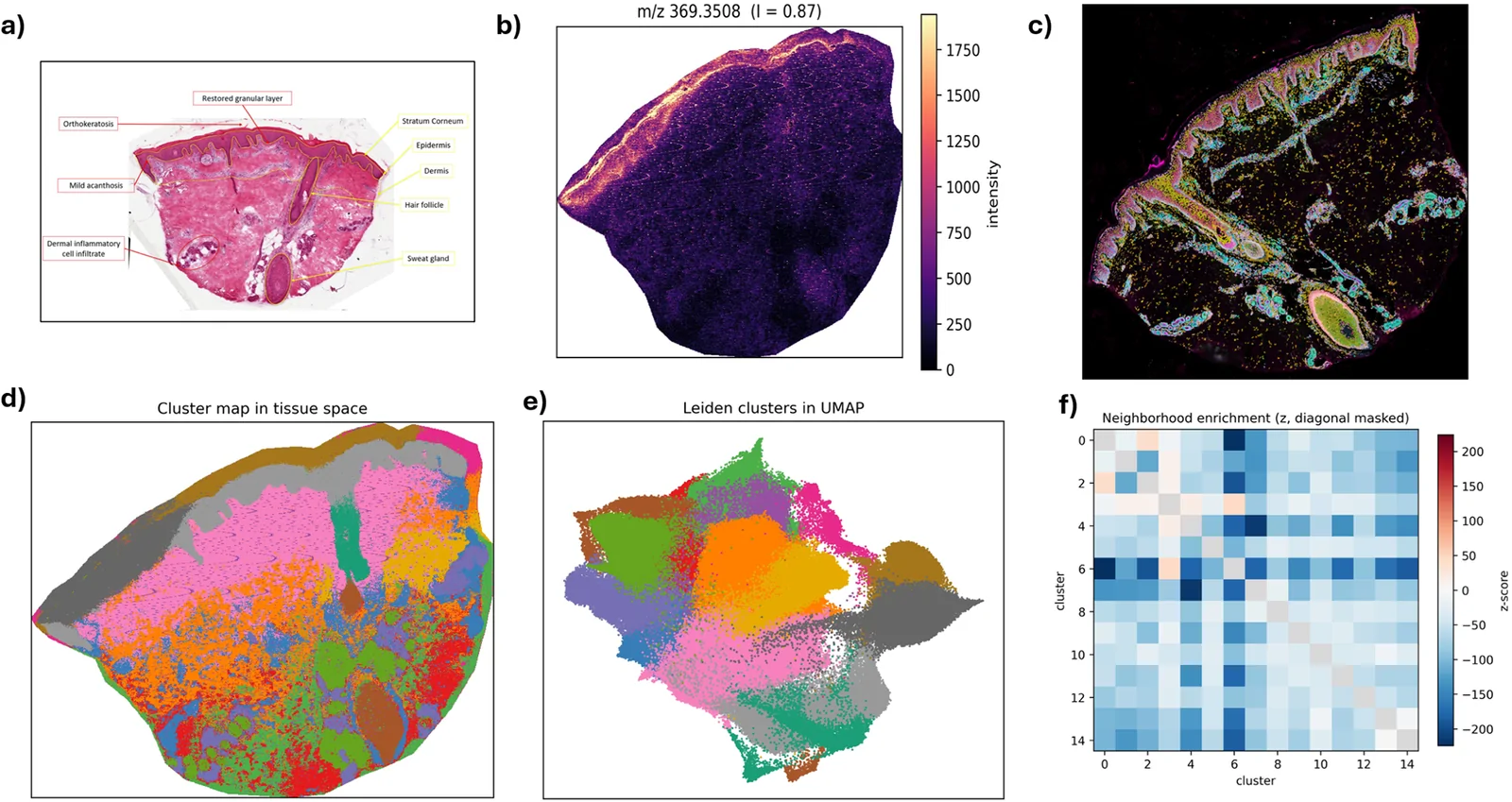
Exploratory analysis on a skin psoriasis MALDI MSI data set. Top row: paired modalities of the multimodal acquisition on the same tissue section. a) Annotated H&E section, with histopathological compartments labeled (yellow: anatomy; red: psoriasis-specific features). b) The strongest spatially structured ion identified by Squidpy’s Moran’s I (*m*/*z* 369.3508, I = 0.87). c) Xenium spatial transcriptomics on the same section. Bottom row: scverse-based analysis of the MSI substrate alone. d) Cluster map rendered in tissue space, which the reader can compare directly with the H&E annotations in panel a). e) UMAP embedding of pixels in spectral space colored by Leiden cluster. f) Neighborhood enrichment matrix (z-score) with the diagonal masked. Spatial registration of the H&E and Xenium modalities to the MSI coordinate system is the natural follow-on once registration tooling is in place; it is not applied in this figure.

Spatial autocorrelation flagged a panel of lipid ions with significant Moran’s I across the tissue. The strongest spatially structured species (*m*/*z* 369.3508, I = 0.87) is shown as the representative ion image in Figure 4. Pixel clustering on the spectral matrix recovered coherent regions corresponding to the histopathologically annotated compartments (epidermis, dermis, sweat gland, hair follicle, dermal inflammatory infiltrate), visible as contiguous clusters in the UMAP embedding and as compact regions in tissue space matching the H&E annotations. The spatial neighborhood enrichment matrix is shown with the diagonal masked: a Leiden community is trivially spatially contiguous, so cluster-vs-self-z-scores otherwise dominate the color scale; masking the diagonal lets the off-diagonal structure span the full dynamic range.

The point of this analysis is not to advance a specific clinical claim, which would require a dedicated study with appropriate patient cohorts and matched controls, but to show the practical consequence of the substrate. The same operations on the same data, before conversion, would have required a custom feature matrix, a custom clustering pipeline, and custom spatial statistics, none of which would have been composed with each other or with future modalities. After conversion, the analysis is a notebook of standard scverse calls, and once registration of the paired Xenium and H&E layers is in place, the same calls extend across all three modalities.

## 4 Discussion

The benchmarks reported here support several observations relevant to the design of the MSI data infrastructure. First, the choice of storage layout has measurable consequences for the dominant analytical operations on the MSI data. Column-oriented Zarr storage delivers an order of magnitude improvement on *m*/*z* range queries, the bottleneck of ion image generation, with comparable or modestly slower performance on per-pixel access. The chunked storage that enables this advantage also incurs a known cost on dispersed small reads,—a property of the OME-NGFF specification that the Zarr v3 sharding feature is explicitly designed to address by grouping multiple chunks into larger files. The current benchmarks use default chunking; tuned chunk sizes and sharding parameters appropriate to MSI data characteristics are expected to close the remaining local pixel access gap without sacrificing cloud-native benefits.

Second, the Thyra conversion engine is deserving of a brief discussion. Conventional approaches build a coordinate list (COO) sparse matrix in memory and convert to compressed sparse column (CSC) format in a second step, with peak memory growing linearly in the number of nonzeros and a two- to three-fold spike during the COO-to-CSC conversion. Thyra avoids both costs by streaming the spectral matrix directly into the output Zarr storage. For data sets up to roughly 30 GB, a two-pass pipeline first counts nonzeros per row to lay out a row-oriented (CSR) sparse index structure, then streams resampled intensities and column indices to disk, with an optional transpose to CSC at the end. For larger data sets, an automatic Precalculated Scatter (PCS) path uses memory-mapped temporary files to scatter intensities into the column-oriented layout in-place, side-stepping the COO build entirely. Both paths keep peak resident memory bounded near 200 MB regardless of data set size, so the same conversion command runs unmodified from gigabyte-scale benchmark slides to the 100-GB-class timsTOF acquisitions that motivate atlas-scale and three-dimensional MSI workflows. This engine is the main engineering contribution to the present work.

Third, and most consequential for the practicing MSI researcher, the scverse readiness benchmark shows that representative analytical operations collapse from hand-built pipelines to short compositions of ecosystem tools when the substrate already carries pixel coordinates and AnnData semantics. This is the practical consequence of choosing a multimodal container rather than an export target. The savings are not in raw I/O; they are in the disappearance of the bookkeeping and feature-matrix code that dominates legacy MSI implementations. Crucially, all three benchmark operations are single-modality: they need only the MSI data, not coregistered histology or transcriptomics, so the benefit is available immediately and is not contingent on future registration tooling.

Several limitations deserve to be mentioned. Conversion correctness has been validated across all supported vendor formats (Section 2.5). The performance benchmarks themselves were conducted on a single representative data set acquired on one instrument geometry; because the benchmark characterizes the shared converted SpatialData representation, which is identical regardless of source vendor, rather than any vendor-specific format, these results are expected to generalize across source vendors. Broader performance characterization across mass analyzers and data set scales is ongoing and will be reported separately. The scverse readiness benchmark uses three operations chosen for their representativeness; alternative operations would yield quantitatively different ratios but, on the basis of the present results, qualitatively similar conclusions. The exploratory analysis of Section 3.4 illustrates the analytical surface that the unified container exposes and is not intended as a primary biological result; full clinical validation, particularly for biomarker discovery and intraoperative use, will require dedicated studies. Mild horizontal striping is visible in low-mass ions of the test data set, reflecting per-line MSI acquisition drift; the same drift is visible in the SCiLS Lab rendering of the same file, confirming that it is an acquisition-level artifact rather than a pipeline artifact. Per-pixel TIC and Pareto normalization reduce but do not eliminate this drift, and we deliberately do not apply ion-level streakiness filtering, since the same striping persists across any MSI analysis tool and is conventionally handled either via spatial postprocessing or at the spectra-acquisition stage, neither of which is part of the analytical operations being benchmarked here.

A final comment concerns ion mobility. Mobility separation is increasingly routine in MSI acquisition, yet carrying the mobility dimension into open formats remains a field-wide challenge: imzML has no widely adopted encoding for mobility-resolved imaging data, and most conversion pipelines, the present Thyra release included, do not carry mobility into the converted representation, retaining only the pixel × *m*/*z* matrix. For peak-picked data, however, the extension is less structural than it first appears: mobility-resolved features remain a two-dimensional pixels-by-features matrix whose features are indexed by (*m*/*z*, mobility) pairs rather than *m*/*z* alone, so the AnnData model accommodates them without a new axis. We consider preserving ion mobility a priority for future work. In the design we have developed, the conversion engine writes mobility-resolved features as an additional table inside the same Zarr store, sharing the pixel index with a default mobility-summed pixel × *m*/*z* table that existing scverse tooling consumes unchanged. Because the sparsity structure, chunk layout, and query patterns of mobility-resolved data differ substantially from those characterized here, the implementation requires dedicated validation and is scheduled for a future Thyra release.

Multimodal extensions of this work are the natural next step. Once MSI lives inside a SpatialData object, registering a paired histology image, an immunofluorescence panel, or a spatial transcriptomics experiment to the same coordinate system makes those modalities directly composable with the scverse-readiness operations demonstrated here. Region-of-interest queries, cross-modal aggregations, and joint spatial statistics that previously required bespoke fusion pipelines are reduced to one or two additional lines on the unified object. The substrate established here is the precondition for those extensions, and the present results show that it is performant and analytically expressive on its own. Registration tools such as EscDat^18^ for cross-modal coordinate alignment, paired with cell-segmentation methods like Segger^19^ on the transcriptomics partner, will make these joint analyses a routine notebook composition rather than a per-experiment engineering task.

Looking further ahead, three lines of work follow from the substrate established here. Atlas-scale and three-dimensional MSI become operationally tractable once data can be partially streamed and analytically queried over the network, with the unit of sharing changing from the full file to the relevant chunk. Clinical and intraoperative deployment requires precisely the FAIR-compliant, cloud-streamable substrate that OME-NGFF and SpatialData provide: bringing MSI into the same data substrate as digital pathology is the integration point for hospital information systems and digital pathology pipelines. Finally, community-aligned development, most concretely through engagement with the scverse community and its proteomics workgroup, will ensure that MSI inherits new tools as they appear rather than waiting for bespoke ports. Plugin-based, community-maintained converters are a sustainable answer to the tooling gravity of the spatial omics ecosystem.

## 5 Conclusions

Thyra brings raw vendor and imzML mass spectrometry imaging data natively into the SpatialData container, removing the format barrier that has kept MSI adjacent to, rather than inside, the spatial omics ecosystem. A two-pass streaming sparse matrix engine writes the spectral matrix directly into a Zarr store with bounded memory regardless of data set size, supporting the 100 GB-class acquisitions that motivate atlas-scale and three-dimensional MSI. Storage and query benchmarks against vendor raw and processed imzML formats on a mouse brain MALDI data set show three- to four-fold storage reductions and an order-of-magnitude improvement in *m*/*z*-range query latency, the bottleneck for ion image generation. A scverse-readiness benchmark using only single-modality operations quantifies the practical consequence of substrate choice: spatial autocorrelation, pixel clustering, and neighborhood enrichment, all standard operations in spatial transcriptomics, run as one-liners on the converted object. Together with a brief exploratory analysis on a skin biopsy from a psoriasis patient, these results show that the conceptual proof of multimodal MSI delivered a decade ago can now be operationalized on commodity hardware and through cloud-native workflows. Multimodal extensions follow naturally once registration tooling is in hand; the present work establishes the foundation on which those extensions will be composed.

## Supplementary Material



## Data Availability

Thyra is available as an open-source project under the MIT license at https://github.com/M4i-Imaging-Mass-Spectrometry/thyra and is installable via PyPI. Scripts to reproduce the storage and query benchmarks of Figure 3, the scverse readiness lines-of-code comparison of Section 3.3, and the exploratory notebook of Figure 4 are provided in the same repository. The storage and analytical-query benchmarks of Figure 3 were performed on the multimodal spatial omics data set generated in this study, available on Zenodo under accession code 10.5281/zenodo.18326569. This deposit contains the following modalities: 1. **Mass Spectrometry Imaging (MSI):** Raw Bruker.d file. 2. **Spatial Transcriptomics:** Raw Xenium output files. 3. **Histology:** High-resolution Brightfield microscopy images (.svs). The skin psoriasis MALDI MSI data set, used only for the exploratory analysis of Section 3.4 (Figure 4), together with its paired Xenium spatial transcriptomics, is available on Zenodo under accession code 10.5281/zenodo.21334918.

## Notes

Average mass spectrum across the tissue, with empty pixels excluded (Figure S1); cluster maps for fwhm-sum integration paired with TIC, median, and Pareto normalization on the curated 267-peak panel (Figure S2); and clustering quality metrics, given as per-pixel neighbor purity and spatial-graph modularity by normalization scheme (Table S1) (PDF)
