# Supplementary material for: Native, Memory-Bounded Conversion of Mass Spectrometry Imaging Data into the SpatialData Ecosystem
Source: J Am Soc Mass Spectrom. 2026 Sep 22;37(10):2379–87. doi: 10.1021/jasms.6c00169 (PMC13651614; doi:10.1021/jasms.6c00169)
Supplement: Supplementary file 1 [file js6c00169_si_001.pdf]

Supporting Information

Native, Memory-Bounded Conversion of Mass Spectrometry Imaging Data into the SpatialData Ecosystem

Theodoros Visvikis[1], Wouter-Michiel Vierdag[2], Luca Marconato[2], Angeliki Birmpili[1], Ron M.A. Heeren[1], Eva Cuypers[1,*]

[1] The Maastricht MultiModal Molecular Imaging (M4I) Institute, Division of Imaging Mass Spectrometry (IMS), Maastricht University, 6229 ER Maastricht, The Netherlands

[2] European Molecular Biology Laboratory, Genome Biology Unit, 69117 Heidelberg, Germany

* Corresponding author: e.cuypers@maastrichtuniversity.nl

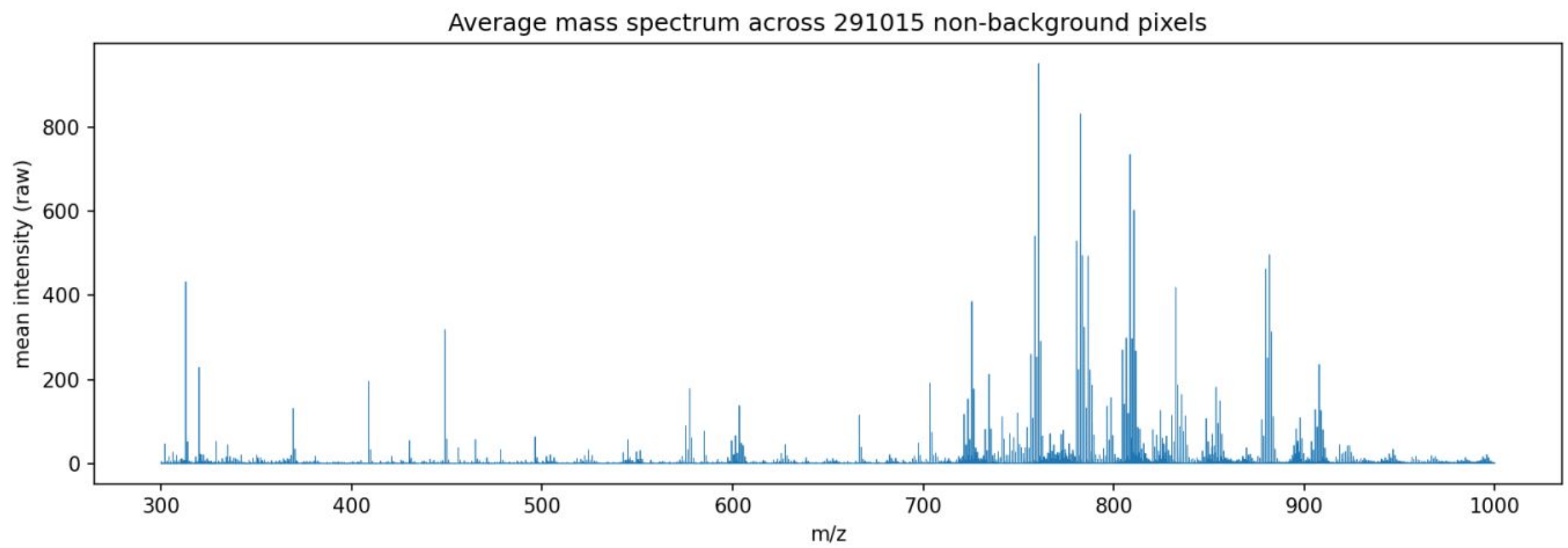

Figure S1: average mass spectrum across the tissue with empty pixels excluded.

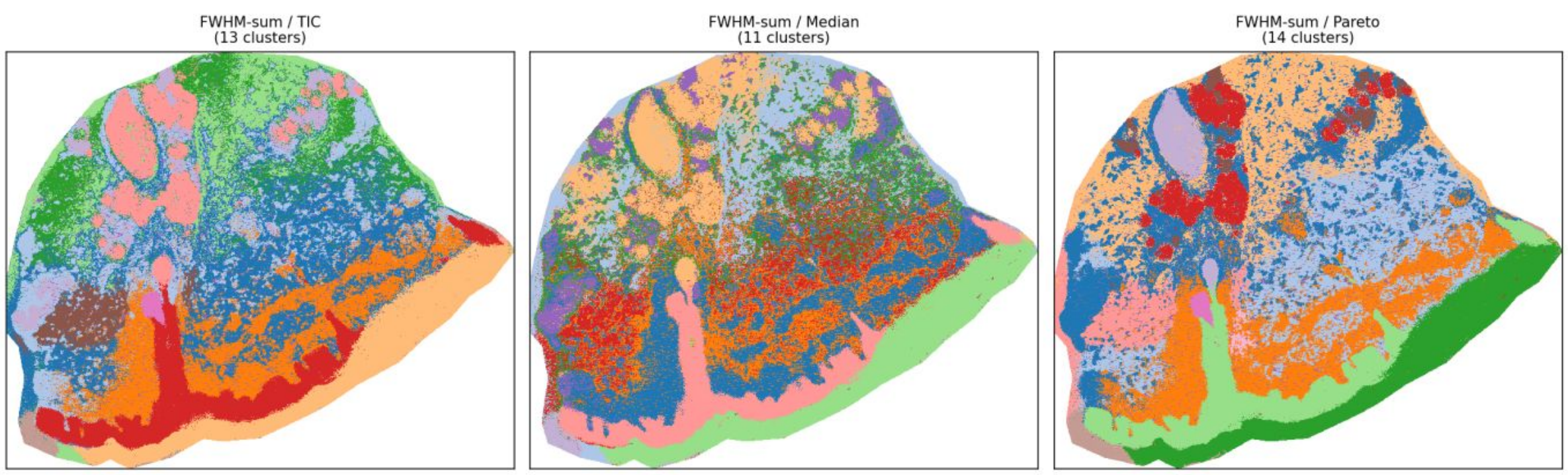

Figure S2: cluster maps for FWHM-sum integration paired with TIC, Median, and Pareto normalization on the curated 267-peak panel, showing why Pareto was chosen.

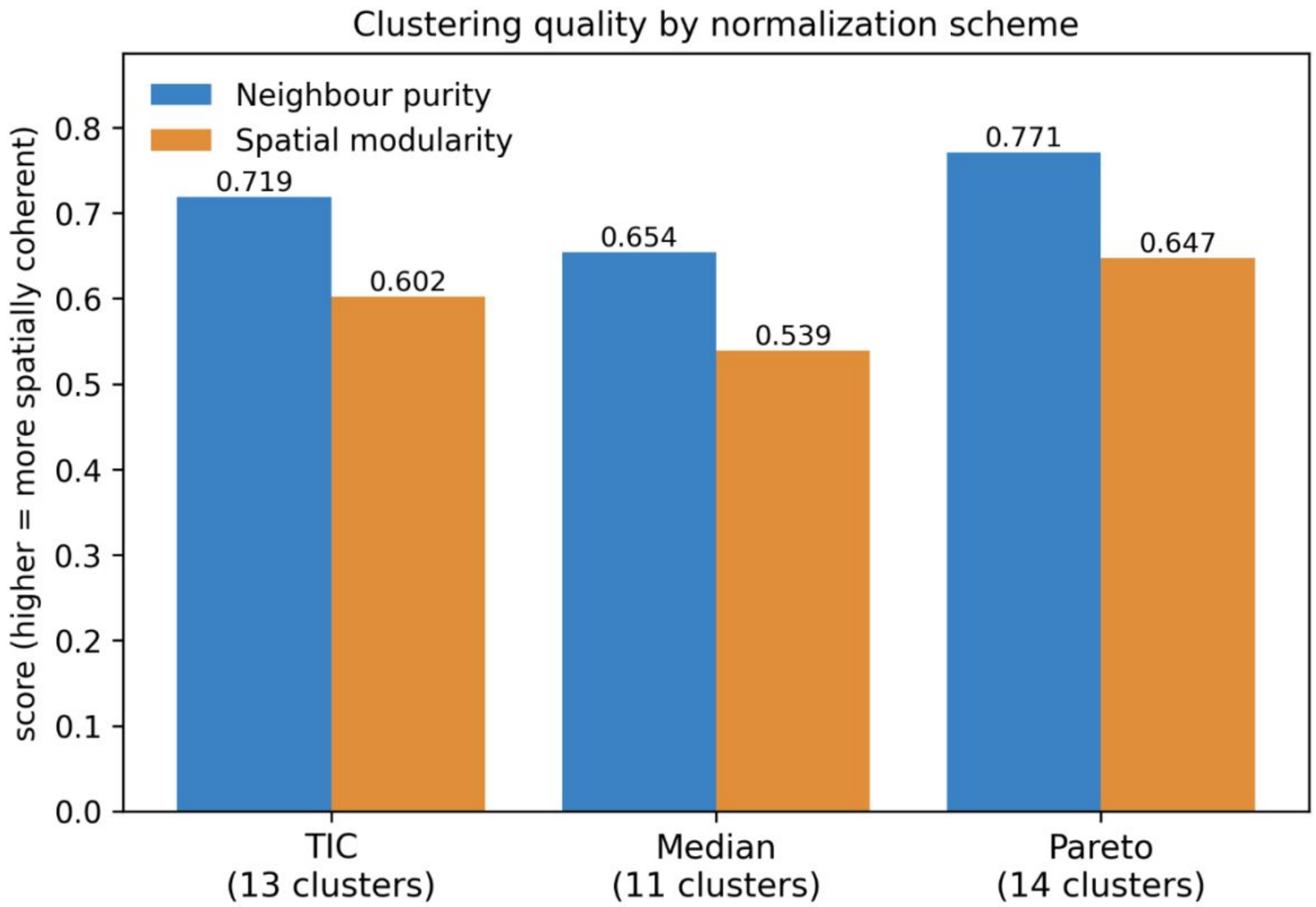

Table S1: clustering quality metrics (per-pixel neighbor purity and spatial-graph modularity) by normalization scheme.
